# Letter to the editor regarding Bahadori and colleagues’ ‘Efficacy of transcranial magnetic stimulation in anorexia nervosa: a systematic review and meta-analysis’ (2025)

**DOI:** 10.1007/s40519-026-01859-z

**Published:** 2026-05-14

**Authors:** Amelia Hemmings, Carla Dirmina, Iain C. Campbell, Ulrike Schmidt

**Affiliations:** 1 Centre for Research in Eating and Weight Disorders, Institute of Psychiatry, Psychology and Neuroscience (IoPPN), King’s College London, London, UK; 2 South London and Maudsley NHS Foundation Trust, Maudsley Hospital, London, UK

Dear Professors Lorenzo Donini and Giovanni Abbate-Daga,

In Volume 30 of Eating and Weight Disorders - Studies on Anorexia, Bulimia and Obesity, in January 2025, Bahadori and colleagues published a systematic review and meta-analysis of the efficacy of transcranial magnetic stimulation (TMS) in anorexia nervosa [1]. This is an important and evolving area of research, aiming in the longer run to broaden treatment options for people with persistent anorexia nervosa, for whom available therapeutic options have not resulted in full and long-lasting recovery.

We have some concerns regarding the methodology of the paper and the conclusions drawn, which we will elaborate on below.

Firstly, the authors report their eligibility criteria: “Original English articles focused on human subjects who underwent TMS for anorexia nervosa. This study encompassed cohorts, cross-sectional studies, case–control studies, case series, and randomized and non-randomized clinical trials” (p. 2). However, three of the included papers are study protocols [2–4]; as such, there are no outcome data reported, and thus we think this makes these papers ineligible for inclusion. These protocol papers were assessed for risk of bias using “the non-randomised studies of interventions (ROBINS-I) tool,” including domains such as bias due to missing data and bias in measurement of outcomes. As protocols do not report outcome data, they cannot be meaningfully nor validly assessed on these metrics. If these papers are to be included, to provide additional information regarding current and upcoming research in this area, it is our opinion that the inclusion criteria should be updated to reflect this, and these papers should not be assessed with the ROBINS-I quality assessment tool.

More broadly, we note that the ROBINS-I was applied across all included studies, including randomised controlled trials (RCTs). ROBINS-I is specifically designed for non-randomised intervention studies and may be inappropriate for assessing RCTs, for which tools such as the Cochrane risk-of-bias tool for randomized trials (RoB 2) are recommended [5]. As such, the use of a single tool applied uniformly across heterogeneous study designs risks mischaracterisation of study quality.

Secondly, there are six publications by Dalton et al. [6–11] which are included as eligible in this review. These are all papers reporting different outcomes (including clinical outcomes at end of trial, longer term open follow-up data, neuroimaging data, qualitative data and food choice task data) from the same study, the TIARA trial. As such, these are not independent samples. The reported effect sizes vary across these publications due to data from different subsets of the overall sample being analysed as a result of attrition over the different assessment time points of the trial. The data nonetheless all originate from the same cohort of 34 participants. The authors treat these publications as independent samples, and by including four of these in their meta-analyses, they quadruple-count their data, and risk artificially inflating the resulting effect.

This issue is particularly relevant for the meta-analysis of the effect of TMS on eating disorder examination questionnaire (EDE-Q) scores, as the TIARA sample is used in four out of the six included studies. Critically, one of these studies, Dalton et al. [10], focuses specifically on the effects of antidepressant medication on rTMS outcomes, where the comparator is antidepressant use rather than sham stimulation, and which does not report the relevant data on the effect of TMS that would be necessary for inclusion in the meta-analysis conducted in this paper.

In addition, the subgroup analysis conducted to examine the effect of TMS session duration on EDE-Q global score highlights the above-mentioned issue of quadruple-counting the TIARA participants more seriously, with the authors concluding: “The results indicated that patients who underwent rTMS sessions lasting more than 20 min experienced better outcomes [31, 32, 33, 34, 35, 38, 64]” (p. 11); the seven papers cited, including one duplicate, all pertain to the TIARA trial, which used session lengths above 20 min; therefore, this repeat-counting will most likely have inflated the standardised mean difference found for this analysis.

We also note that the statistical analysis figures given in the results section appear to be different than those reported in the abstract. For example, in the abstract, the effect of rTMS on body mass index (BMI) change is reported as: “SMD: −0.025, 95% CI −0.0505 to −0.005, *P*-value = 0.045” (p. 1), and in the results section this is reported as: “SMD = −0.255, 95% CI −0.505 to −0.005, and *P*-value = 0.045” (p. 3). In addition, in our opinion, the Forest plots and Funnel plots are ambiguous, as the effect on BMI (reported as an overall increase in BMI after rTMS), is graphically represented as a negative standardised mean difference, and the effect on EDE-Q (reported as an overall decrease in EDE-Q global score after rTMS), is represented as a positive SMD value.

Furthermore, we feel there was insufficient consideration given in the current meta-analysis to the heterogeneity present in the included papers, in particular, the different target regions for stimulation. Different rTMS targets, intended to cause different, specific, neural changes, may have different effects on anorexia symptoms. We are concerned about the validity of a meta-analysis comparing heterogeneous neural targets.

The paper draws the conclusion that “The findings revealed a significant increase in body mass index (BMI) following TMS (SMD: −0.025, 95% CI −0.0505 to −0.005, *P*-value = 0.045),” and “The analysis exhibited a significant decrease in EDE-Q score after TMS (SMD: 0.634, 95% CI 0.349–0.919, *P*-value < 0.001). Subgroup analysis based on TMS session duration indicated that the effect size of TMS on EDE-Q score is more pronounced when the session duration exceeds 20 min” (p. 1). Due to the issues we have outlined, particularly the multiplication of overlapping samples, the use of evidence drawn from inappropriate comparator conditions, and the pooling of studies targeting heterogeneous regions, we are concerned that the conclusions drawn may not be appropriate or valid.

## Data Availability

No datasets were generated or analysed during the current study.
